# Editorial: Building on a Firm Foundation

**DOI:** 10.1289/ehp.11131

**Published:** 2008-01

**Authors:** Hugh A. Tilson

**Affiliations:** E-mail: EHPEditor@niehs.nih.gov

On 2 January 2008, I began serving as editor-in-chief of *Environmental Health Perspectives* (*EHP*). In doing so, I follow in the footsteps of the many editors who came before me—James Burkhart, Thomas Goehl, George Lucier, and Gary Hook. Since the start of the journal in 1972, these individuals have guided the development of *EHP* to be one of the world’s premier environmental health science publications. In 2007, *EHP* published 12 issues containing more than 300 peer-reviewed research articles (i.e., commentaries, reviews, mini-monographs, meeting and workshop reports, environmental medicine, children’s health), as well as numerous editorials and letters. Each issue also contained a news section, which included articles on emerging research themes; investigative articles on a wide range of national and international topics; analytical articles on legal, regulatory, public policy, and social aspects of environmental health science; and articles describing new discoveries or approaches in research, remediation, monitoring, and public health policy. *EHP* also supports numerous outreach activities, including the publication of a Chinese-language edition and a student edition. The cumulative result of the hard work of the previous editors of *EHP* is reflected in an impact factor of 5.86, which is the highest for journals publishing in the area of environmental sciences. Clearly, the challenge for the next editor-in-chief is to build on the successes of the previous editors. In the future, the journal must be ready to address emerging areas in environmental health science and adapt to changes in how science is communicated to a diverse audience.

In plotting a course for the evolution of *EHP*, there are several principles that are critical for its ultimate success: independence, transparency, balance, and recognition of emerging themes. Two of the most important of these are independence and transparency. Although it is true that *EHP* receives generous support from the National Institute of Environmental Health Sciences (NIEHS), it is necessary that the content, scope, and direction of the journal not be influenced by NIEHS leadership. As the *EHP* editor-in-chief, I must have the full responsibility for directing and managing all aspects of the publication. The journal must also operate in an open and transparent manner. Guidelines for submission and publication of papers and news articles must be clear and followed consistently. Papers meeting conditions for review—relevance to human health, adequate sample sizes, novelty of information, impact on the field—must be evaluated by fair and impartial associate editors and reviewers. Reasons for rejection or acceptance of articles must be clearly articulated to the authors.

A balance of differing opinions offers the journal credibility and depth. This should be reflected in the makeup of the associate and editorial review boards and in the content of the science and news articles. It is also important that the journal publish news and research about emerging themes in environmental health science. Over the last several years, *EHP* has published many excellent epidemiologic studies on air pollution, metals, and persistent bio-accumulative toxicants. The journal will continue to provide a forum wfor this type of research. *EHP* is also in a position to attract papers involving multidisciplinary or integrated approaches to complex environmental issues, including cumulative risk, community risk, systems biological approaches to hazard identification, exposure science, source-to-effect modeling, and approaches to evaluate the effectiveness of regulatory decisions. *EHP* also has a long history of publishing toxicologic studies. Looking forward, multidisciplinary research papers elucidating modes of action or critical steps in biologic pathways that result in toxicity would be consistent with this tradition. Another emerging trend in environmental health science is the reevaluation of the default process to evaluate human health risk after exposure to environmental agents. Approaches involving analysis of uncertainty in risk assessment and the application of alternative risk assessment models need to be aired in a public forum.

Also important to the success of the journal will be its emphasis on outreach to developing countries and to young people interested in a career in environmental health science. Finally, it is clear that *EHP*’s continued success will depend on the innovative application of the Internet to facilitate communication of science news and results to diverse audiences.

I believe that adhering to the principles of independence, transparency, balance, and recognition of emerging themes—in conjunction with a timely and efficient review process—will lead to a wider audience for the journal and an enhanced impact on the field. I look forward to working with the talented and committed staff at *EHP* to ensure the reality of this vision.

## Figures and Tables

**Figure:**
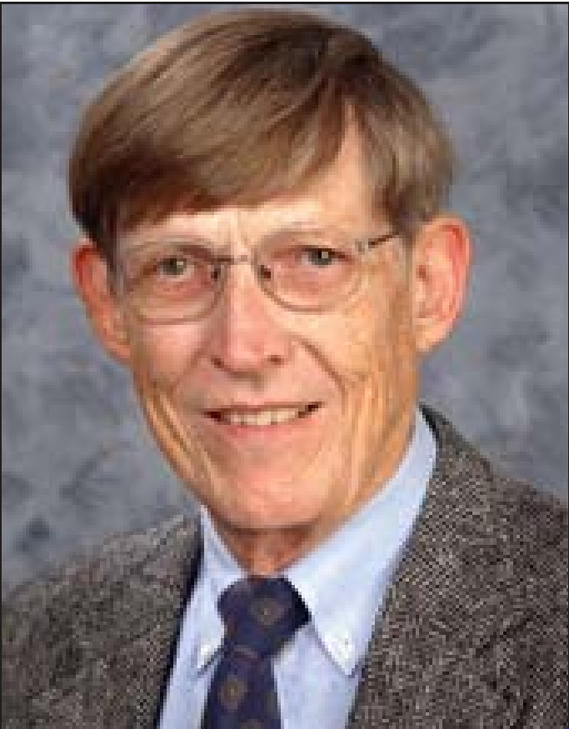
Hugh A. Tilson

